# Secretory structures in *Mandevilla venulosa* (Müll.Arg.) Woodson (Apocynaceae): morphoanatomy and histochemical characterization

**DOI:** 10.1007/s00114-026-02120-4

**Published:** 2026-06-01

**Authors:** Luís Henrique Bueno, Fabricio Antônio de Morais, Miller Melo Sanches, Karina Lucas Barbosa Lopes

**Affiliations:** 1 Departamento de Biologia Vegetal, Universidade Federal de Viçosa UFV, Viçosa, 36570-900 Minas Gerais Brazil; 2 Instituto Federal de Educação Ciência e Tecnologia do Sul de Minas Gerais - Campus Muzambinho, Muzambinho, Brazil

**Keywords:** Colleters, Laticifers, Rocky outcrops, Tuberous root, Secretion, Latex

## Abstract

*Mandevilla venulosa* is an endemic species of rocky outcrops in high-altitude fields of the Serra da Pedra Branca, located in Caldas, Minas Gerais, Brazil, within the Atlantic Forest phytogeographic domain. Secretory structures are common in Apocynaceae and may provide useful anatomical and taxonomic information. This study aimed to describe the anatomy and histochemistry of colleters and laticifers in *M. venulosa*. Fresh vegetative samples were collected and processed using standard microscopy techniques and subjected to histochemical tests. The colleters observed correspond to the standard type, composed of a central parenchymatic region surrounded by a uniseriate palisade secretory epidermis, with dense cytoplasm and a thin cuticle. During the secretory phase, they release a viscous and translucent fluid. The secretion contains polysaccharides, mucilage, pectins, and proteins, compounds associated with protection against desiccation and microbial activity. Articulated and anastomosing laticifers occur in all vegetative organs, forming early in development and producing latexes with organ-specific chemical compositions: a milky, hydrophobic latex in aerial parts (rich in lipids, terpenoids, alkaloids, and phenolics) and a yellow, hydrophilic latex in the tuberous root (containing polysaccharides, mucilage, proteins, and phenolics). Colleters are involved in protecting developing regions and preventing the desiccation of young organs. On the other hand, laticifers contribute to herbivory control due to their phenolic composition. This study contributes to the anatomical knowledge of secretory structures in *Mandevilla*. Notably, it provides the first evidence that latex composition may vary among different organs within the same species, highlighting the relevance of including subterranean structures in future studies to better understand latex diversity in the genus.

## Introduction

Apocynaceae is one of the largest angiosperm families, comprising approximately 400 genera and about 5,000 species, distributed mainly in subtropical regions (Endress et al. 2014, Endress 2018). The family is notable for the diversity of its secretory structures - including colleters, laticifers, nectaries, trichomes, and osmophores - which are involved in the production of a wide variety of secondary metabolites, including alkaloids, phenolics, and terpenoids (Monteiro and Demarco 2017; Souza et al. 2021; Salomé et al. 2023; Figueiredo et al. 2025). The presence, anatomical features, and chemical composition of these structures hold significant taxonomic importance; for instance, the distribution and number of colleters are used as diagnostic characters to delimit groups within the family (Woodson and Moore 1938; Appezzato-da-Glória and Estelita 2000). These secretory structures may serve as diagnostic characters for infrageneric delimitations within *Mandevilla* Lindl. In this genus, the distribution of colleters along the midrib on the adaxial surface of the leaf blade, as well as their number and position at the base of the sepals, represent important traits for distinguishing clades such as the “*exothostemon*” and “*mandevilla*” clades (Simões et al. 2006; Morales et al. 2022). These structural variations thus provide relevant morpho-anatomical support for the circumscription of taxa and for recognizing distinct evolutionary lineages within the family.

Within the subfamily Apocynoideae, the genus *Mandevilla* stands out as the largest Neotropical group, comprising approximately 200 species (Morales et al. 2022). It is distributed from Mexico and the southern United States, through the West Indies, to Argentina (Alvarado-Cárdenas and Morales 2014). The genus exhibits considerable morphological diversity, including shrubs, herbs, lianas, and epiphytes, and it occurs in a range of habitats such as coastal sandbanks, sandy formations in the Amazon basin, Andean forests, rocky fields (campos rupestres), dry forests, wetlands, and savannas (Simões et al. 2004; Morales 2007, 2009, 2011). Furthermore, the genus tends toward endemism in inselbergs and quartzitic outcrops (Morales and Kollmann 2019, 2020; Morales et al. 2022; Arantes et al. 2024; Morales 2025). In Brazil, *Mandevilla* is represented 79 species, 51 of which are endemic (Flora e Funga do Brasil 2025). These species can be easily recognized by racemose inflorescences, anthers with truncate bases, and stylar heads bearing five longitudinal projections (Endress 2018).

*Mandevilla venulosa* (Müll.Arg.) Woodson is an erect subshrub with glabrous to pubescent branches, white flowers with a yellowish interior, and opposite, decussate, sessile or subsessile leaves, which are ovate-elliptic or oblong-elliptic in shape, with an obtuse or slightly acuminate apex, cordate base, and subcoriaceous to coriaceous texture (Vasconcellos and Gouvea 1993). Endemic to the rocky outcrops of high-altitude fields in southern Minas Gerais and at the border with São Paulo state, *M. venulosa* is considered vulnerable, as listed in the Red List of the Flora of São Paulo (CNCFLORA 2012). The ecosystems of high-altitude fields, rocky outcrops, and inselbergs are subject to harsh environmental conditions, including low temperatures, strong winds, and shallow soils (Neri et al. 2017). These conditions impose high levels of stress on plants, making them vulnerable to environmental changes (Hermant et al. 2013; Leão et al. 2014; Cordeiro 2017).

The Serra da Pedra Branca (SPB), located in the municipality of Caldas, southern Minas Gerais, exemplifies this scenario, ranging from 1,100 to 1,780 m and predominantly composed of high-altitude fields (Rezende et al. 2013). The region is recognized as an area of conservation importance for the flora (Drummond et al. 2005). However, these environments face increasing anthropogenic pressures, particularly due to granite mining activities (Rezende et al. 2013). Mining activities represent a major threat to high-altitude fields and rocky outcrops. About one-third of active mining areas worldwide are located in intact regions of conservation importance, and around 75% are situated in areas with high endemism and ecological relevance (Morales et al. 2022).

Secretory structures are widely distributed in plants and perform several protective and physiological functions. Among them, colleters are secretory organs that produce a viscous exudate composed mainly of mucilage and other hydrophilic or lipophilic compounds (Thomas 1991; Tullii et al. 2013; Teixeira et al. 2022). This secretion lubricates and protects developing buds and may also inhibit the proliferation of fungi and phytophagous insects (Fahn 1979, 1990; Thomas 1991; Demarco 2017; Ribeiro et al. 2017; Tullii et al. 2023). In addition to colleters, laticifers are structures that synthesize and store latex and are widely distributed throughout the plant body in various families, functioning mainly in defense, especially against herbivores (Metcalfe 1967; Fahn 1979; Mahlberg 1993; Ramos et al. 2019). Laticifers are an almost universal feature in Apocynaceae (Metcalfe 1967). However, no studies have yet investigated the anatomy and histochemistry of colleters and laticifers in *M. venulosa*.

Therefore, this study aimed to characterize the colleters and laticifers present in the vegetative organs of *M. venulosa* and to describe the chemical nature of their exudates. We also discuss the potential taxonomic relevance of these secretory structures within the genus.

## Materials and methods

**Fig. 1:**
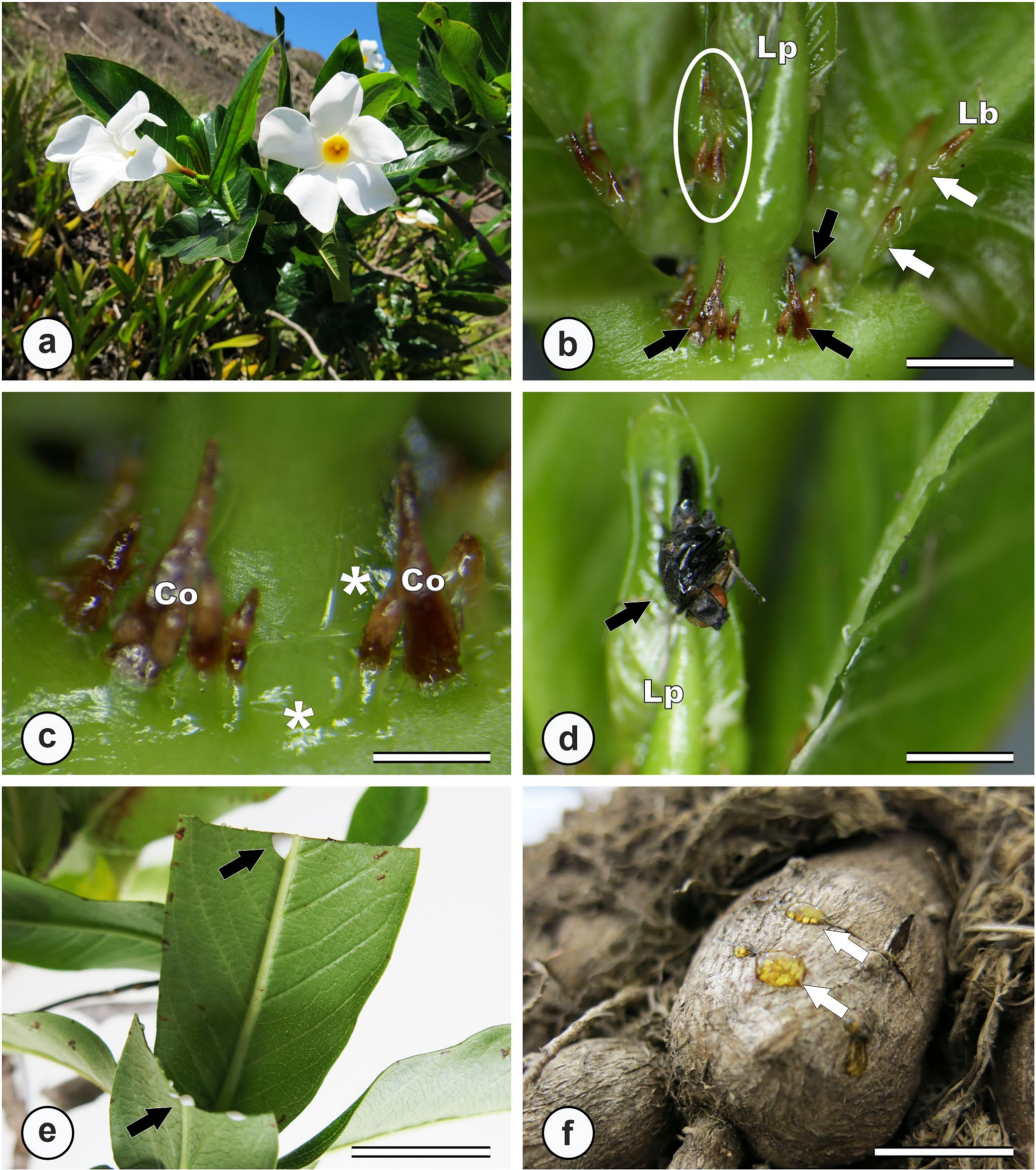
External morphology of the vegetative organs of *M. venulosa* and exudation of substances by secretory structures. **a**. General view of the species growing on a rocky outcrop. **b**. Colleters are visible at the leaf base (white arrows), in intra- and interpetiolar regions (black arrows), and on leaf primordia (circle). **c**. Translucent viscous secretion (*) produced by senescent interpetiolar colleters; note the brown coloration at the colleter apex extending toward the base. **d**. Hymenopteran trapped in colleter exudate, with visible insect wings (black arrow). **e**. White viscous latex exuding from leaves after mechanical injury. **f**. Yellowish viscous latex exuding from the tuberous root following injury. Co = colleter; Lp = leaf primordium; Lb = leaf base. Scale bars: b, d = 2 mm; c = 1 mm; e, f = 2 cm

Samples of *M*. *venulosa* were collected from five individuals at Serra da Pedra Branca (SPB), (Fig. 1a) in the municipality of Caldas, southern Minas Gerais (21°58′–21°55′S, 46°24′–46°22′W). Fertile branches of the species were collected in the field (Morais FA 1; 2). Voucher specimens were deposited in the Ander Fredrik Regnell Herbarium of the Poços de Caldas Botanical Garden (Minas Gerais, Brazil) under the records AFR72 and AFR73.

For anatomical analysis under light microscopy, shoot apices, nodal regions, fully expanded leaves (midrib and margin), stems, and roots of *M. venulosa* were fixed in FAA 50 (formaldehyde, glacial acetic acid, 50% ethanol) for 48 h and then stored in 70% ethanol (Johansen 1940). Samples were dehydrated through an ethanol series and embedded in methacrylate resin (Historesin, Leica Instruments). Transverse and longitudinal sections were obtained using a rotary microtome with automatic advancement (model RM2155, Leica Microsystems Inc.), equipped with disposable steel blades. Serial Sects.  (5–7 μm thick) were stained with toluidine blue at pH 4.0 (O’Brien and McCully 1981), and slides were mounted in synthetic resin (Permount).

Histochemical tests on vegetative shoot apices and nodal regions were performed on historesin-embedded samples sectioned with the rotary microtome. The following reagents were used: Lugol (Johansen 1940) for starch detection; Ruthenium Red (Johansen 1940) for pectins and mucilage; periodic acid-Schiff reagent (O’Brien and McCully 1981) for total polysaccharides; Xylidine Ponceau (O’Brien and McCully 1981) for total proteins; ferric chloride (Johansen 1940) for phenolic compounds; Fresh samples of the leaf midrib were hand-sectioned in the longitudinal plane and subjected to histochemical tests, like as Sudan IV (Pearse 1968) for lipids, Wagner’s reagent (Furr and Mahlberg 1981) for alkaloids; Nadi reagent (David and Carde 1964) for essential oils and oleoresins; and Oil Red (Pearse 1968) for rubber. The reference material was used as the control.

The anatomical analyses and documentation of permanent and semi-permanent botanical slides were conducted using a light microscope (Olympus BX43) equipped with an image capture system (Olympus U-TV1X-2, T7, Tokyo, Japan) at the Microscopy Laboratory of IFSULDEMINAS – Muzambinho campus. Observations and photographic documentation of histochemical tests were carried out using a light microscope (AX70TRF; Olympus Optical, Tokyo, Japan) equipped with an image capture system (AxioCam HRr; Zeiss, Göttingen, Germany) at the Plant Anatomy Laboratory of the Federal University of Viçosa. Images of colleters were obtained using a stereomicroscope (Olympus SZX7 TR30, Japan) and captured with a camera (Olympus Evolt E-330, Japan) at Plant Tissue Culture Laboratory II of the Institute of Biotechnology Applied to Agriculture (BIOAGRO), Federal University of Viçosa.

## Results

### Anatomical structure and histochemical profile of colleters in *M. venulosa*

In *M. venulosa* (Fig. 1a), colleters were observed in various regions of the plant, including leaf primordia, petioles (with both intrapetiolar and interpetiolar occurrence), the leaf blade base, and nodal regions (Fig. 1b-c). During the secretory phase, colleters were light green and released a viscous translucent exudate (Fig. 1b-c). Upon completion of secretory activity, these structures entered senescence, characterized by a gradual darkening that began at the apex and progressed toward the base (Fig. 1c). Even after secretion ceased, the colleters persisted on the vegetative structures. Hymenoptera were found stuck on the colleter exudates at the shoot apex of *M. venulosa* (Fig. 1d).

**Fig. 2:**
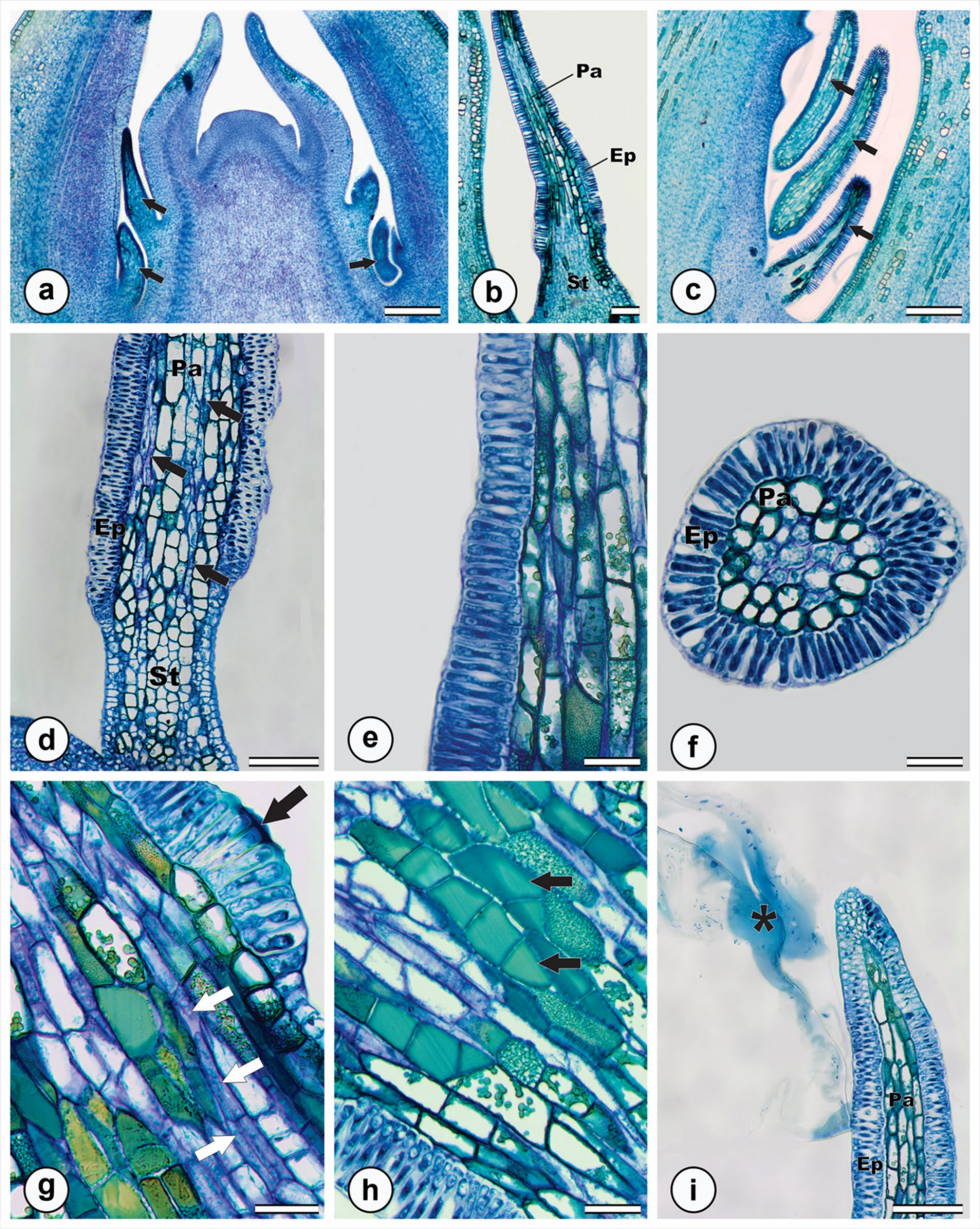
Anatomical characterization of *M. venulosa* colleters stained with toluidine blue. **a**. Vegetative shoot apical meristem showing the location of colleters (arrows = colleters). **b**. General view of a standard-type colleter. **c**. Colleters located on leaf primordia. **d**. View of the multiseriate stalk (St) with non-secretory epidermal cells; note the presence of laticifers (arrow). **e**. Detail of the colleter secretory epidermis, note the dense cytoplasm. **f.** Transverse section of the colleter. **g**. Secretion accumulated in the secretory epidermis (black arrow) and laticifers (white arrow). **h**. Idioblasts in the parenchymatic region of the colleter (arrows). **i**. Colleter secretion exuding to the external environment (*). (Ep = epidermis; Pa = parenchyma; St = stalk). Scale bars: 200 μm (a, c); 100 μm (b, d, i); 40 μm (e-h)

Colleters consisted of a central parenchymatous region surrounded by a uniseriate secretory epidermis (Fig. 2a-c). The stalk, which varied in length, comprised two to twenty layers of rectangular parenchymatous cells covered by a non-secretory epidermis composed of cuboidal to rectangular cells (Fig. 2c-d). The secretory epidermis consisted of palisade-like cells with thin cell walls and cuticle and dense cytoplasm (Fig. 2e-f). The secretion accumulated in the intercellular spaces of the secretory epidermis (Fig. 2g). Laticifers and phenolic idioblasts were observed throughout the parenchymatous region of the colleters (Fig. 2d, g-h). The secretion was released externally without evidence of cuticle rupture or distension (Fig. 2i).

**Fig. 3:**
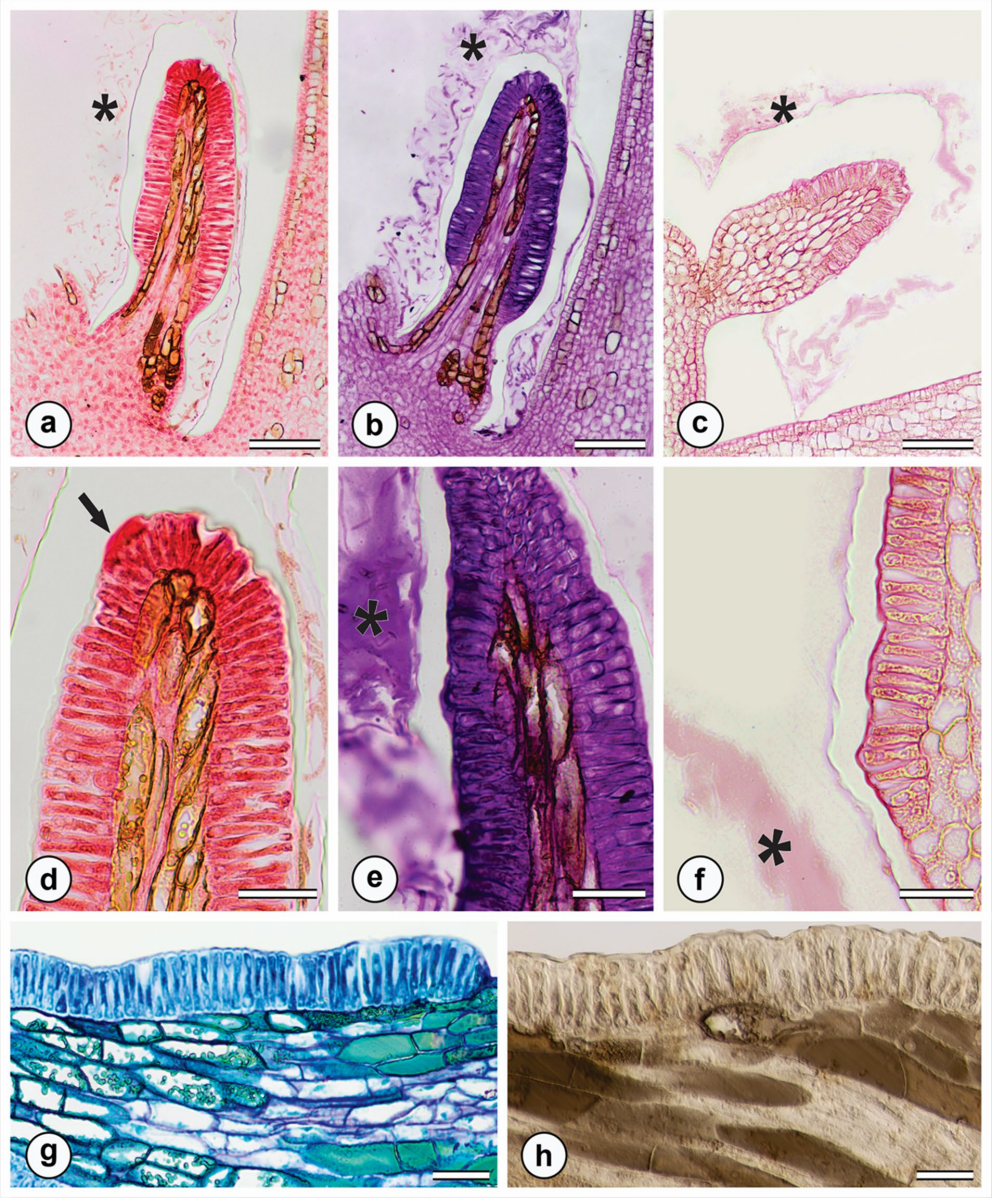
Histochemical tests on colleters of *M. venulosa* in longitudinal sections, using Xylidine Ponceau (a, d), periodic acid–Schiff (PAS) reagent (b, e), ruthenium red (c, f), toluidine blue (g), and ferric chloride (h). **a**. Detection of total proteins in the colleter, with visible secretion (*). **b**. Detection of total polysaccharides, highlighting secreted material (*). **c**. Presence of mucilages and pectins in the secretion (*). **d**. Accumulation of protein-rich secretion at the colleter apex (arrow). **e**. Polysaccharide-rich secretion released to the external environment (*). **f**. Mucilage identified in the external secretion (*). **g**. Phenolic compounds stained green in the subepidermal and parenchymatic regions. **h**. Phenolic compounds stained dark brown in the subepidermal and parenchymatic regions. Scale bars: 100 μm (a-c); 40 μm (d-h)

Histochemical analyses revealed the presence of proteins, polysaccharides, pectins/mucilage and phenolic compounds in both the secretion and the secretory cells of the colleters (Fig. 3a-h). Starch and lipids were not detected in the secretion (Table 1).

**Table 1:** Histochemical characterization of the secretory structures in M. venulosa

Metabolite class	Test	Reagent	Result		
			Laticífer Leaf	Laticífer Tuberous root	Colleter
Polysaccharides	Starch	Lugol	-	-	-
	Total polysaccharides	PAS	-	+	+
	Pectins / mucilage	Ruthenium red	-	+	+
Lipids	Lipids	Sudan IV	+	-	-
Terpenoids	Essential oils/resins	NADI reagent	+	NA	-
	Rubber	Oil Red	+	-	-
Proteins	Total proteins	Coomassie Brilliant Blue	+	+	NA
		Xylidine Ponceau	+	+	+
Phenolics	Total phenolics	Toluidine Blue	+	+	+
	Phenolic compounds	Ferric chloride	+	-	-
Alkaloids	Alkaloids	Wagner reagent	+	NA	NA

“+” = positive reaction; “−” = negative reaction; “NA” = not assessed

### Anatomical structure and histochemical profile of laticifers in *M. venulosa*

**Fig. 4:**
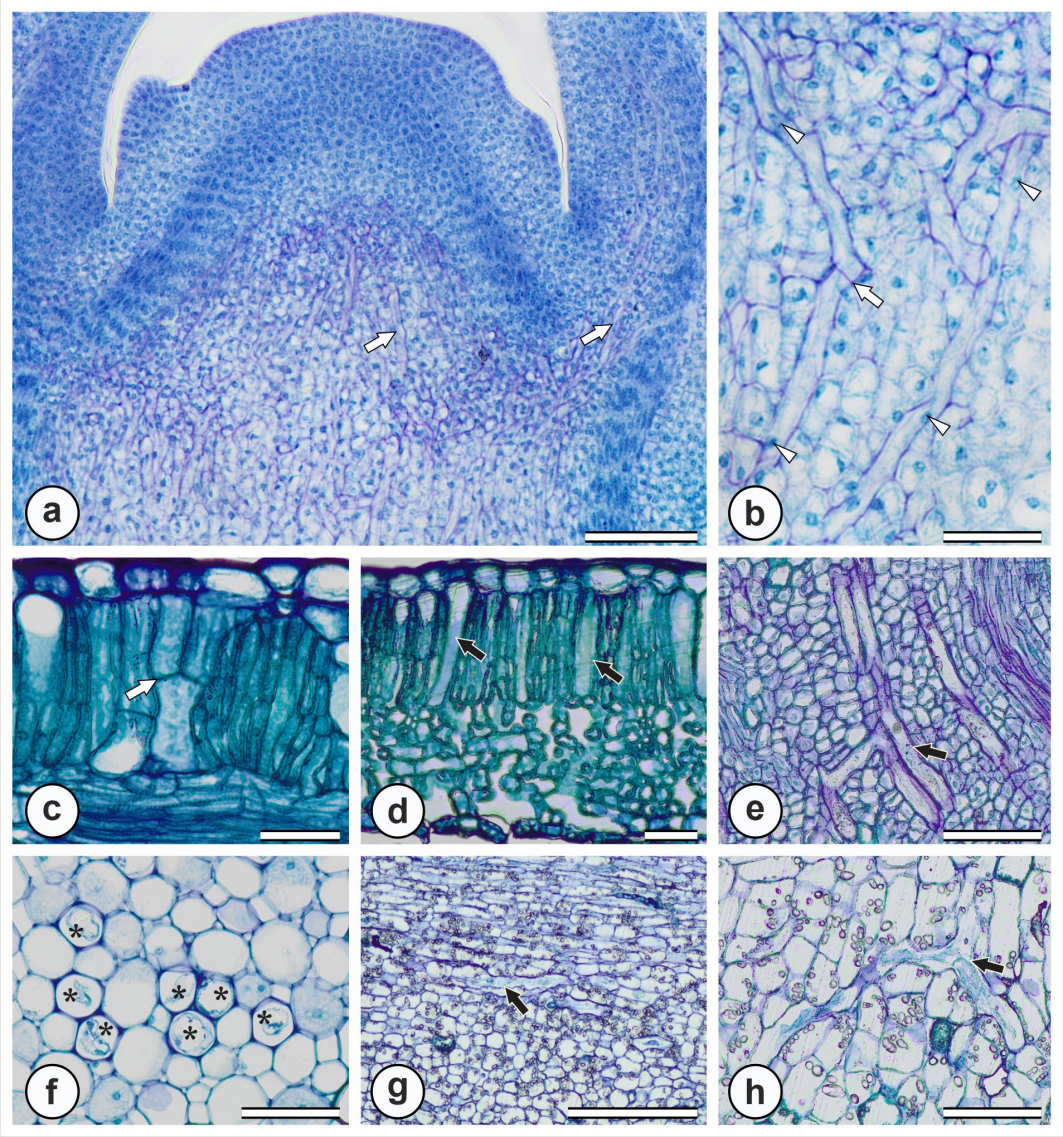
Distribution of laticifers in the vegetative organs of *M. venulosa*. **a**. Laticifers in the meristem of the stem apex (black arrows). **b**. Anastomosis between two laticifers (black arrow) with evident nuclei (arrowhead). **c**. Laticifers in the palisade parenchyma of the leaf blade, connected by anastomoses (white arrow). **d**. Laticifers located in the spongy and palisade parenchyma (white arrow). **e**. Branched laticifers in the stem cortex. **f**. Laticifers in the stem medulla (*), with thicker cell walls compared to adjacent cells. **g**. Laticifers near the root cortex (black arrow). **h**. Branched laticifer in the cortical region of the root (black arrow). Scale bars: 100 μm (a, e, h); 40 μm (b, c, d, f); 200 μm (g)

When the latex of *M. venulos*a was exuded, it appeared as a viscous white fluid in aerial organs (Fig. 1e), whereas in the underground system, it presented as a viscous yellow fluid (Fig. 1f). In the shoot apex, laticifers originated from cells of the ground meristem and procambium, which are already differentiated while most of the surrounding tissues are still in the meristematic phase. These laticifers elongated vertically along the longitudinal axis of the plant (Fig. 4a-b). They were formed by the sequential addition of cells whose walls dissolved rapidly, leading to extensive fusion of adjacent cells and resulted in continuous laticifers with conspicuous spherical nuclei (Fig. 4a). The laticifers of *M. venulosa* were articulated anastomosing and gave rise to branched laticifers, which can be observed in meristematic regions (Fig. 4b).

Laticifers were present in all vegetative organs of the plant: leaves (Fig. 4c-d), stems (Fig. 4e-f), and the tuberous root (Fig. 4g-h). In the leaf blade, laticifers occurred in the palisade parenchyma, sometimes reaching the epidermis, and in the spongy parenchyma associated with vascular bundles, branching through intercellular spaces (Fig. 4c-d). In the stem, laticifers were observed in both the cortex and the pith (Fig. 4e-f). The walls of laticifers in the stem pith are thicker than those of surrounding cells (Fig. 4e). In the roots, laticifers occurred predominantly in the cortex (Fig. 4g). These structures often branched, forming Y-shaped configurations (Fig. 4e, h).

**Fig. 5:**
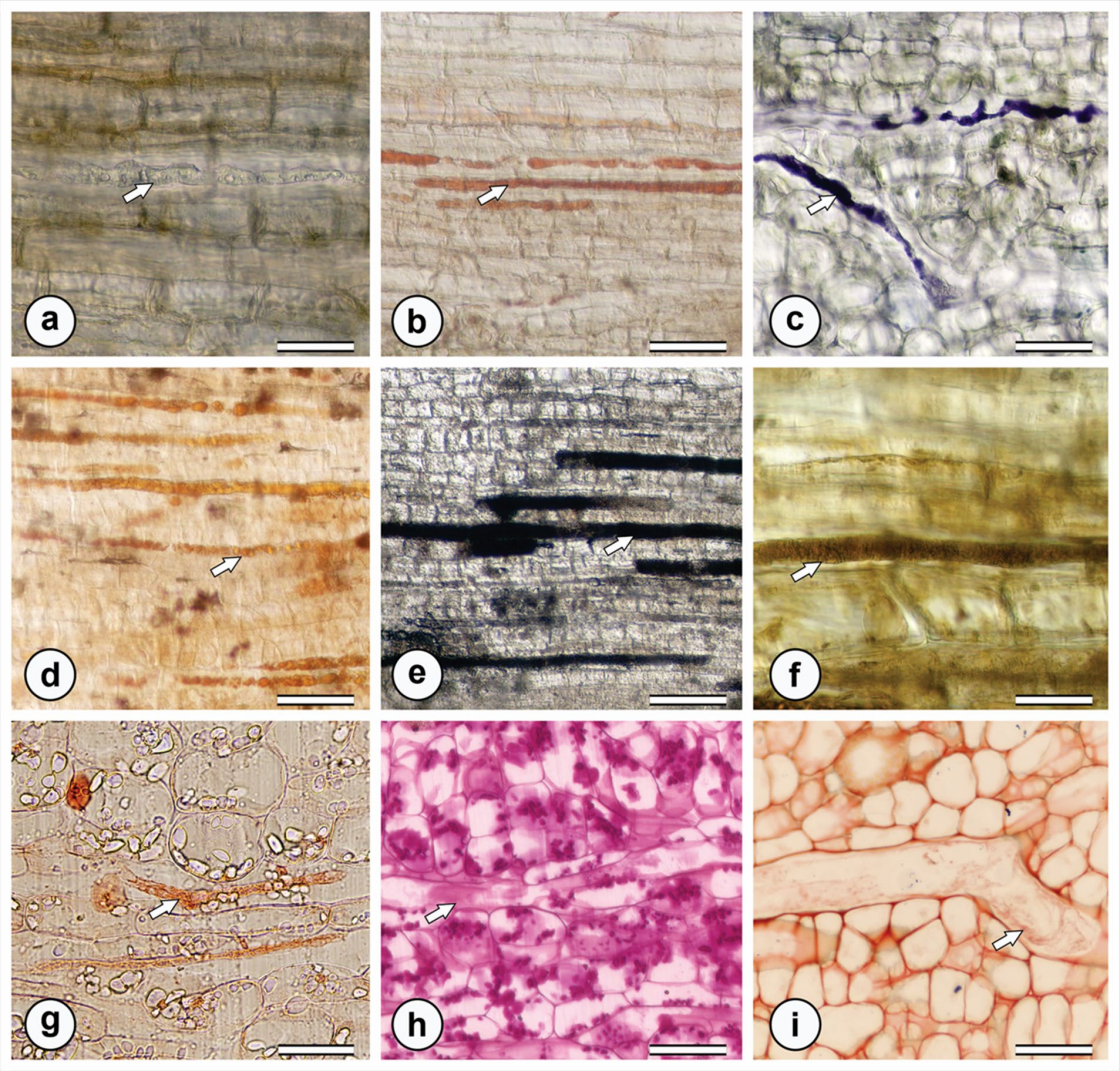
Histochemical tests for the identification of compounds present in the latex of the leaf (a-f) and the tuberous root (g-i) of *M. venulosa*. **a**. Laticifer located in the midrib without reagent treatment. **b**. Lipid droplets inside the laticifers detected by positive reaction with Sudan III. **c**. Positive reaction with Nadi reagent, indicating the presence of oils/resins in a branched laticifer. **d**. Rubber detected by staining with Oil Red. **e**. Presence of tannins evidenced by positive reaction with ferric chloride. **f**. Alkaloids detected by positive reaction with Wagner’s reagent. **g**. Proteins detected by positive reaction with Xylidine Ponceau. **h**. Polysaccharides identified by positive reaction with PAS reagent. **i**. Mucilage/pectins detected by positive reaction with ruthenium red. White arrow indicate laticiferous secretion. Scale bar: 50 μm

Histochemical tests revealed that the milky latex found in aerial organs is hydrophobic, while the yellow latex of the tuberous root is hydrophilic (Table 1). The milky latex showed positive reactions for lipophilic compounds, terpenoids, alkaloids, and phenolic compounds, but tested negative for polysaccharides, mucilage, and proteins (Table 1). In its natural color in anatomical sections, was observed as white viscous latex in the leaf and crystalline yellow latex in tuberous root (Fig. 5a). The latex from aerial organs tested positive for lipids (Fig. 5b), oil-resins (Fig. 5c), rubber (Fig. 5d), total phenolic compounds (Fig. 4c, e), and alkaloids (Fig. 5f). The latex from underground organs tested positive for phenolic compounds, proteins, polysaccharides, and pectins/mucilage, and negative for lipids and rubber. The latex in the tuberous root showed positive results for total phenolics (Fig. 4h), proteins (Fig. 5g), polysaccharides (Fig. 5h), and mucilage (Fig. 5i).

## Discussion

### Structure, secretion and possible functions of colleters in *M. venulosa*

The colleters of *M. venulosa* are present on leaf primordia, the petiole, and at the base of the leaf blade. The distribution of these structures holds taxonomic relevance within Apocynaceae, as colleters can occur on various plant organs, including the leaf blade and petiole, cotyledons, bracts, bracteoles, calyces, and corollas (Woodson and Moore 1938; Fahn 1979; Thomas and Dave 1989; Thomas 1991; Canaveze and Machado 2016; Demarco 2017). In *M. venulosa*, the colleters correspond to the “standard type” as defined by Lersten (1974). This is the most common type within the genus, having been reported in *M. illustris* (Vell. Woodson), *M. pohliana* (Stadelm.) A.H. Gentry (= *M. velutina* (Mart. ex Stadelm.) Woodson), and *M. tenuifolia* (J.C. Mikan Woodson) (Appezzato-da-Glória and Estelita 2000; Silva 2015). Standard-type colleters have also been widely reported in Apocynaceae (Thomas and Dave 1989; Rio et al. 2002; Martins et al. 2010).

The secretory epidermal cells of *M. venulosa* colleters exhibit dense cytoplasm, indicating high metabolic activity, a feature also reported for colleters in other Apocynaceae species (Martins et al. 2010; Silva 2015; Ribeiro et al. 2017, 2021). In addition, the presence of a non-secretory stalk is frequently described in colleters of the family (Appezzato-da-Glória and Estelita 2000; Martins et al. 2010; Capelli et al. 2017; Güven 2025).

Vascularization in these secretory structures does not follow a consistent pattern across Apocynaceae and also varies within *Mandevilla*. Vascularized colleters have been reported in several members of the family (Woodson and Moore 1938; Dave et al. 1987; Appezzato-da-Glória and Estelita 2000; Rio et al. 2002; Martins et al. 2010). However, the absence of vascular tissue has also been described in closely related species, such as *M. tenuiflora* (Silva 2015), as observed in *M. venulosa*. According to Appezzato-da-Glória and Estelita (2000), vascularization in *Mandevilla* colleters may depend on the proximity of these structures to vascular bundles in the associated organ. This variability suggests that the presence or absence of vascular tissue is not a consistent taxonomic character for the genus but rather a trait that may vary among species and organs (Demarco et al. 2006).

Colleter secretions are generally composed predominantly of mucilage (Thomas 1991), although lipophilic substances are frequently reported in the family (Thomas and Dave 1989; Appezzato-da-Glória and Estelita 2000; Castro and Demarco 2008). A heterogeneous secretion, comprising mucilage, proteins, lipids and phenolic compounds, is effective in protecting meristematic regions from desiccation, fungal proliferation and herbivorous insects (Ribeiro et al. 2017). In *M. venulosa*, the colleter secretion contains mucilage, polysaccharides, and proteins. Mucilage is generally the primary component of colleter exudates, or at least one of the most significant (Rio et al. 2002; Mayer et al. 2011; Martins 2012; Cardoso-Gustavson et al. 2014; Canaveze and Machado 2016; Demarco 2017; Ribeiro et al. 2017, 2021). Its high water-retention capacity prevents desiccation of meristematic tissues (Fahn 1979; Simões et al. 2004; Ribeiro et al. 2021). High-altitude fields are characterized by water scarcity, intense wind exposure, shallow soils, wide temperature fluctuations, and high solar radiation (Safford 1999a, b), conditions under which *M. venulosa* occurs. In this context, the polysaccharides present in colleter secretions may play a crucial role in lubricating vegetative apices and maintaining the integrity of young tissues under environmental stress. These compounds contribute to water retention and provide viscosity to the secretion (Toneli et al. 2005; Cassola et al. 2019). In addition, hydrated polysaccharides can form a dense matrix that acts as a physical barrier against microorganisms (Cassola et al. 2019). Proteins found in colleter secretions have been associated with defense against microbial and insect attack. For instance, colleters of *Bathysa* (Rubiaceae) produce antifungal proteins (Miguel et al. 2006).

In *M. venulosa*, colleter secretion is released externally without rupture of the cuticle a mechanism also reported in other Apocynaceae species (Thomas and Dave 1989; Appezzato-da-Glória and Estelita 2000; Martins et al. 2010; Silva 2015; Demarco 2017; Ribeiro et al. 2021). However, ultrastructural studies are needed to clarify the exact processes involved in this type of secretion.

Other secretory structures, such as laticifers and phenolic idioblasts, are commonly found within colleters, particularly in the parenchymatous region. This occurrence has been reported in several genera of Apocynaceae (Thomas and Dave 1989, 1991; Appezzato-da-Glória and Estelita 1997, 2000; Demarco 2008; Silva 2015). Phenolic compounds play important roles in plant survival across diverse environments, contributing to protection against herbivores and pathogens, antioxidant activity, tolerance to environmental stress, and functioning as light filters that help modulate radiation reaching internal tissues (Fahn 1979; Simões et al. 2004; Kumar et al. 2020; Tak et al. 2023; Emus-Medina et al. 2023). In *M. venulosa*, the presence of phenolic idioblasts in colleters may contribute to the protection of meristematic regions and leaf primordia.

Following the secretory phase, colleters undergo senescence. At this stage, a gradual color change from the apex to the base can be observed, with the structures becoming dark brown, a pattern previously reported for colleters in Apocynaceae (Thomas 1991; Appezzato-da-Glória and Estelita 2000; Miguel et al. 2010, 2023; Tullii et al. 2013; Lopes-Mattos et al. 2015; Canaveze and Machado 2016; Ribeiro et al. 2021; Gonçalves et al. 2022). Senescence in colleters generally begins soon after secretion ceases (Paiva 2009; Fernandes et al. 2016; Ribeiro et al. 2017). However, studies have also reported overlapping secretory and senescent phases, with early senescence signs appearing even during secretion (Gonçalves et al. 2022). In *M. venulosa*, colleters do not display synchronized development a phenomenon also observed in other species (Miguel et al. 2010, 2023; De Paiva Pinheiro et al. 2019). Further research is necessary to better understand the senescence process in *M. venulosa* colleters, particularly the mechanisms governing phase transitions and the triggers of senescence.

Plants established on rocky outcrops often grow directly on exposed rocks where soils are shallow or absent (Meirelles et al. 1999) and are subject to extreme temperature fluctuations, low relative humidity, and high solar radiation (Safford 1999a, b; Neri et al. 2017). Species inhabiting these environments generally exhibit adaptive strategies linked to desiccation tolerance (Porembski and Barthlott 2000; Benites et al. 2007) and are particularly vulnerable to climate change (Neri et al. 2017). Since *M. venulosa* occurs in high-altitude fields, xeric habitats shaped by strong environmental pressures, the presence of mucilage likely helps prevent dehydration of developing organs, while phenolic idioblasts may act as feeding deterrents, reducing herbivory in an already stressful environment.

### Functional and taxonomic significance of laticifers in *M. venulosa*

Laticifers in *M. venulosa* are found in both aerial and subterranean organs. The presence of latex is a widely distributed feature in the Apocynaceae family and is considered a recurring anatomical marker within the group (Metcalfe 1967; Mahlberg 1993). Although latex is universally present, there is considerable variation in the classification of these secretory structures. In Apocynaceae, both articulated and non-articulated laticifers occur (Metcalfe 1967; Mahlberg 1993; Demarco and Castro 2008; Canaveze and Machado 2016). Until the early 21st century, the prevailing view was that only non-articulated laticifers were present in the family. However, subsequent studies have shown that articulated laticifers, especially the anastomosing type, are widely distributed and frequently occur in different genera of Apocynaceae (Demarco et al. 2006; Demarco and Castro 2008; Lopes et al. 2009; Canaveze and Machado 2016; Gama et al. 2017; Souza et al. 2021; Marques et al. 2023; Salomé et al. 2023; Figueiredo et al. 2025).

Anatomical heterogeneity regarding the type of laticifer is common in the genus, and the presence of non-articulated laticifers in both aerial and subterranean organs agrees with observations in *M. illustris* and *M. pohliana* (Appezzato-da-Glória and Estelita 1997). On the other hand, *M. atroviolacea*, a species also found in rocky outcrops of high-altitude fields, possesses articulated laticifers (Lopes et al. 2009). A re-evaluation of laticifer classification in Apocynaceae is necessary to clarify whether laticifers traditionally described as non-articulated might actually be articulated laticifers with rapidly dissolving transverse walls (Demarco et al. 2006; Lopes et al. 2009). The early formation of laticifers in *M. venulosa*, in the procambium and ground meristem of shoot apical meristems, has also been reported for *M. atroviolacea* (Lopes et al. 2009) and other Apocynaceae species (Lopes et al. 2009; Gama et al. 2017; Gonçalves et al. 2018; Souza et al. 2021; Figueiredo et al. 2025).

Latex is composed of an emulsion containing various types of substances, such as polyisoprene hydrocarbons (rubber), starches, proteins, fatty acids, oils, sugars, alkaloids, sterols, tannins, as well as numerous enzymatic proteins, protease inhibitors, and several allergens (Konno 2011). In *M. venulosa*, the chemical composition of latex varies depending on the organ analyzed, for instance, milky latex in the aerial parts and yellow latex in the tuberous root. The latex found in the leaves is rich in lipophilic compounds, terpenoids, alkaloids, and phenolics, and resembles that of other species in the genus, such as *M. atroviolacea* (Lopes et al. 2009), M. *illustris*, and *M. pohliana* (Appezzato-da-Glória and Estelita 1997), although the latter have not been tested for the presence of terpenoids and alkaloids.

Despite the existence of morphological and anatomical studies of vegetative and underground organs (Appezzato-da-Glória 2003; Appezzato-da-Glória and Estelita 1997, 2000; Duarte and Larrosa 2011), few investigations have addressed the composition of latex in tuberous roots or xylopodia (Lopes et al. 2009). In the present study, we found that the yellow latex from the tuberous root of *M. venulosa* is hydrophilic in nature, consisting mainly of phenolic compounds, proteins, polysaccharides, and mucilage. The histolocalization of these and other compounds, as reported in previous studies (Lopes et al. 2009; Appezzato-da-Glória and Estelita 1997), highlights the need for further chemical characterization of latex in this genus, particularly for potential biotechnological applications. Notably, this study provides the first evidence that latex composition may vary among different organs within the same species, emphasizing the importance of including subterranean structures in future investigations to better understand the chemical diversity and functional roles of latex in the genus.

Beyond its biotechnological potential, latex-producing laticifers are secretory structures that play crucial ecological roles in plant interactions with both biotic and abiotic environmental factors (Fahn 1979). Laticifers contribute to protection against predators such as chewing insects and microorganisms, and they serve as a sealing agent, as latex coagulates upon exposure to air (Fahn 1979; Farrell et al. 1991; Agrawal and Konno 2009; Konno 2011; Lopes et al. 2009). The chemical compounds in latex can deter herbivory through toxicity or anti-nutritional effects, and the sticky consistency hinders the movement of herbivorous insects (Agrawal and Konno 2009; Konno 2011).

Approximately 10% of angiosperms exude latex when injured (Farrell et al. 1991), with this proportion reaching 20–35% among tropical American species (Lewinsohn 1991), compared to other latex-producing plants worldwide (Farrell et al. 1991). Latex production can also be influenced by environmental factors such as light intensity, water deficit, soil fertility, and herbivory pressure (Agrawal and Konno 2009). In addition to deterring herbivores and pathogens, laticifers may also contribute wound healing and to water retention (Freitas et al. 2010; Ramos et al. 2019; Teixeira et al. 2020). Therefore, the evolutionary success of some plants in diverse environments is frequently attributed to the presence of laticifers, due to the protection they provide to both their vegetative and reproductive organs (Konno 2011; Ramos et al. 2019). These functions are particularly relevant for *M. venulosa*, which occurs on rocky outcrops in high-altitude fields characterized by nutrient-poor soils, abundant rock fragments, and intense climatic stress, conditions that increase the risk of injury.

## Conclusion

This study provides the first anatomical and histochemical description of colleters in *M. venulosa*, revealing that they correspond to the standard type, which is the only one reported so far for the genus. Colleters were mainly observed in young and meristematic organs and released a viscous secretion containing mucilage, polysaccharides, and proteins. Laticifers were classified as articulated and anastomosing and occurred in all vegetative organs of the species, with early differentiation in meristematic tissues. Histochemical tests revealed the presence of metabolites such as lipids, oils and resins, tannins, alkaloids, and rubber in the latex.

In addition, this study reports differences in latex composition between aerial organs and the tuberous root, indicating organ-specific variation in the chemical profile of the secretion. These results expand the anatomical and histochemical knowledge of secretory structures in Mandevilla and contribute to the understanding of structural diversity within Apocynaceae.

## Data Availability

No datasets were generated or analysed during the current study.
